# Glucocorticoid resistance of migration and gene expression in a daughter MDA-MB-231 breast tumour cell line selected for high metastatic potential

**DOI:** 10.1038/srep43774

**Published:** 2017-03-06

**Authors:** Ebony R. Fietz, Christine R. Keenan, Guillermo López-Campos, Yan Tu, Cameron N. Johnstone, Trudi Harris, Alastair G. Stewart

**Affiliations:** 1 Department of Pharmacology and Therapeutics, University of Melbourne, Parkville, Victoria 3010, Australia; 2 Health and Biomedical Informatics Centre, University of Melbourne, Parkville, Victoria 3010, Australia; 3 Peter MacCallum Cancer Centre, East Melbourne, Victoria 3002, Australia

## Abstract

Glucocorticoids are commonly used to prevent chemotherapy-induced nausea and vomiting despite a lack of understanding of their direct effect on cancer progression. Recent studies suggest that glucocorticoids inhibit cancer cell migration. However, this action has not been investigated in estrogen receptor (ER)-negative breast tumour cells, although activation of the glucocorticoid receptor (GR) is associated with a worse prognosis in ER-negative breast cancers. In this study we have explored the effect of glucocorticoids on the migration of the ER-negative MDA-MB-231 human breast tumour cell line and the highly metastatic MDA-MB-231-HM.LNm5 cell line that was generated through *in vivo* cycling. We show for the first time that glucocorticoids inhibit 2- and 3-dimensional migration of MDA-MB-231 cells. Selection of cells for high metastatic potential resulted in a less migratory cell phenotype that was resistant to regulation by glucocorticoids and showed decreased GR receptor expression. The emergence of glucocorticoid resistance during metastatic selection may partly explain the apparent disparity between the clinical and *in vitro* evidence regarding the actions of glucocorticoids in cancer. These findings highlight the highly plastic nature of tumour cells, and underscore the need to more fully understand the direct effect of glucocorticoid treatment on different stages of metastatic progression.

Glucocorticoids are used extensively and best known as anti-inflammatory and immunosuppressive agents. However, over 30 years ago, the glucocorticoid dexamethasone was reported to be effective in preventing chemotherapy-induced nausea and vomiting^1^,^2^. Since that time, glucocorticoids have also been shown to prevent postoperative^3^,^4^ and radiotherapy-induced^5^ nausea and vomiting. Thus, glucocorticoids constitute one of the major classes of drugs that are considered first-line anti-emetic agents, together with 5HT_3_ receptor antagonists, such as ondansetron, tachykinin NK_1_ receptor antagonists, such as aprepitant, and dopamine D_2_ receptor antagonists, such as metoclopramide^6^. Despite the common use of glucocorticoids as anti-emetics in cancer patients receiving chemotherapy, the mechanism of action of this effect remains unclear. Several mechanisms have been proposed^7^ including: decreasing the inflammatory response from chemotherapy or radiation^8^,^9^,^10^; direct actions on the solitary tract nucleus in the central nervous system which receives emetogenic inputs from the afferent sympathetic and parasympathetic nervous systems^11^; effects on key mediators/receptors in emetic pathways such as 5HT and NK receptors^12^,^13^,^14^; and effects on the hypothalamic-pituitary-adrenal (HPA)-axis^15^. Rapid glucocorticoid actions related to increased levels of endocannabinoid CB1 ligands may also play a role in the antiemetic effect of glucocorticoids^16^,^17^. Furthermore, there is a surprising lack of characterisation of the direct effect of glucocorticoids on the development and progression of different cancers.

At a molecular level, glucocorticoids act through the glucocorticoid receptor (GRα, herein referred to as GR) and predominantly work by modulating gene transcription, although non-genomic mechanisms are increasingly being reported^18^. In the absence of ligand, the GR is principally located in the cytoplasm in an inactive multi-protein complex^19^. Upon ligand binding, the GR undergoes a conformational change whereby the complexed proteins dissociate and nuclear localisation signals are exposed, promoting rapid nuclear translocation. Once in the nucleus, the ligand-bound GR is able to: initiate transactivation of genes containing glucocorticoid response elements (GREs); repress transcription of genes containing a negative GRE (nGRE); and trans-repress the activity of other transcription factors such as activator protein-1 (AP-1) and nuclear factor kappa B (NF-κB)^20^,^21^. The number of genes modulated by glucocorticoid administration varies depending on cell type and context^20^,^21^. However, in some circumstances over 10% of the entire genome can be regulated following a single dose of glucocorticoid^22^.

Breast cancer is the second most common cancer worldwide, and the most common cancer in females^23^. Breast cancer mortality is primarily due to metastatic spread of the tumour to secondary sites, such as bone and lung. Functional GR is expressed in almost all cell types, including breast tumour cells and the surrounding stromal tissue^24^,^25^, highlighting the imperative to understand what direct effects glucocorticoid use may have on breast tumour cell biology. Unlike in lymphoid cells where glucocorticoids cause cell death, glucocorticoids inhibit apoptosis of epithelial cell types^26^ including breast tumour cells^27^. This promotion of cell survival has been proposed to interfere with the cytotoxic effects of chemotherapy^28^,^29^, although there is a lack of clinical studies to support this idea^30^. Glucocorticoids may also decrease cell invasion by reducing tissue permeability^31^. Importantly, activation of the GR has been shown to be associated with a worse prognosis in estrogen receptor (ER)-negative breast cancer^32^,^33^. This is particularly significant, since ER-negative tumours are often intrinsically more metastatic than ER-positive tumours^34^. Furthermore, few treatment or prevention strategies are available for patients with ER-negative breast cancer^35^. Recent studies have shown that glucocorticoids can inhibit migration of neuronal precursor cells^36^, A549 lung adenocarcinoma cells^37^, MCF10A breast epithelial cells^38^, and estrogen-receptor positive T47D breast cancer cells^39^. However, the effect of glucocorticoids on estrogen-receptor negative breast tumour cell migration is yet to be established.

In this study, we have explored the effect of glucocorticoids on the migration of the ER-negative MDA-MB-231 human breast tumour cell line. Cell migration is a critical component of metastasis, allowing cells to intravasate and travel via the circulation to seed in secondary organs, forming metastases. We have therefore also examined the changes in migratory phenotype and glucocorticoid sensitivity that occur following selection of cells with high metastatic potential, using the MDA-MB-231-HM.LNm5 cell line. This cell line was generated by *in vivo* passaging of tumour cells which has been demonstrated to be a useful method to select for highly metastatic cell lines^40^.

## Results

### Glucocorticoids inhibit the migration of the ER-negative MDA-MB-231 human breast tumour cells

**Figure 1:**
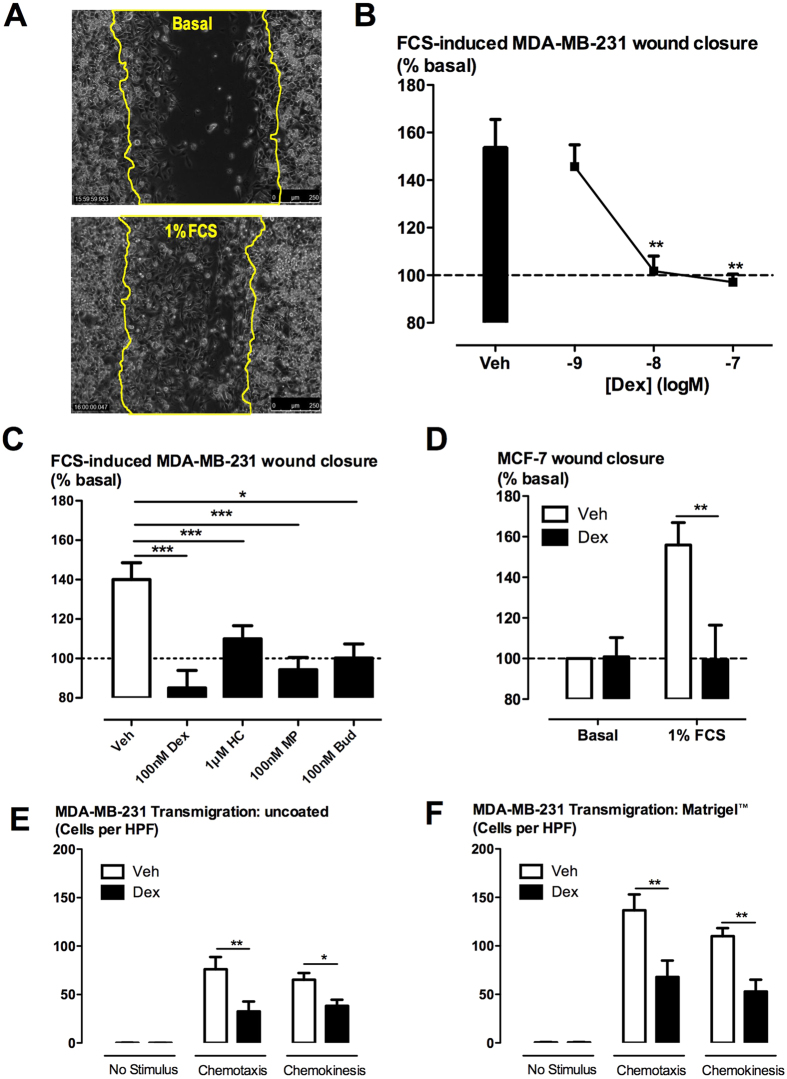
Effect of glucocorticoids on 2-dimensional migration of ER-negative MDA-MB-231 and ER-positive MCF-7 breast tumour cells. Serum-starved MDA-MB-231 cells (**A**,**B**,**C**) and MCF-7 cells (**D**) were scrape-wounded and then pre-incubated with glucocorticoids (dexamethasone, Dex; hydrocortisone, HC; methylprednisolone, MP; budesonide, Bud) for 30 min prior to the addition of FCS (1% v/v) to promote cell migration. Wound infiltration was measured by the change in grey-value using ImageJ/FIJI software (**A**). The extent of wound closure 15 h after FCS addition is expressed as a percentage of basal and presented as mean ± SEM of n = 4 independent experiments. Chemotactic and chemokinetic migration of serum-stared MDA-MB-231 cells was observed using uncoated (**E**) and Matrigel™-coated (**F**) transwell inserts in a modified Boyden chamber assay. Dexamethasone was added to both compartments 30 min before FCS (1% v/v) that was added to both compartments to stimulate chemokinetic migration, or bottom compartment only to stimulate chemotactic migration. After 20 h, migrated cells were stained using Diff-Quick™ and visualised by light microscopy at a magnification of 400×. Data are expressed as the number of migrated cells per high powered field (HPF, 400×) and presented as mean ± SEM of n = 3 independent experiments. *P < 0.05, **P < 0.01, ***P < 0.001 from repeated measures one-way ANOVA with Dunnett’s post-hoc test (**B**,**C**) or two-way ANOVA with Bonferroni post-hoc test (**D**,**E**,**F**).

Fetal calf serum at a sub-maximal concentration of 1% (Supplementary Figure S1) was used as a stimulus for MDA-MB-231 cell migration in the scrape wound healing assay (Fig. 1A). Dexamethasone concentration-dependently inhibited FCS-induced MDA-MB-231 cell migration with complete inhibition observed at 10 nM Dex (Fig. 1B). Dexamethasone at 100 nM had no effect on cell proliferation as measured by direct cell enumeration and by the resazurin assay (Supplementary Figure S2). Other glucocorticoids, including methylprednisolone (100 nM), budesonide (100 nM) and hydrocortisone (1 μM), similarly inhibited MDA-MB-231 cell migration (Fig. 1C). Furthermore, dexamethasone fully inhibited the migration of the non-invasive ER-positive MCF-7 breast tumour cell line (Fig. 1D).

### Glucocorticoids inhibit 3-dimensional MDA-MB-231 human breast tumour cell migration and invasion

**Figure 2:**
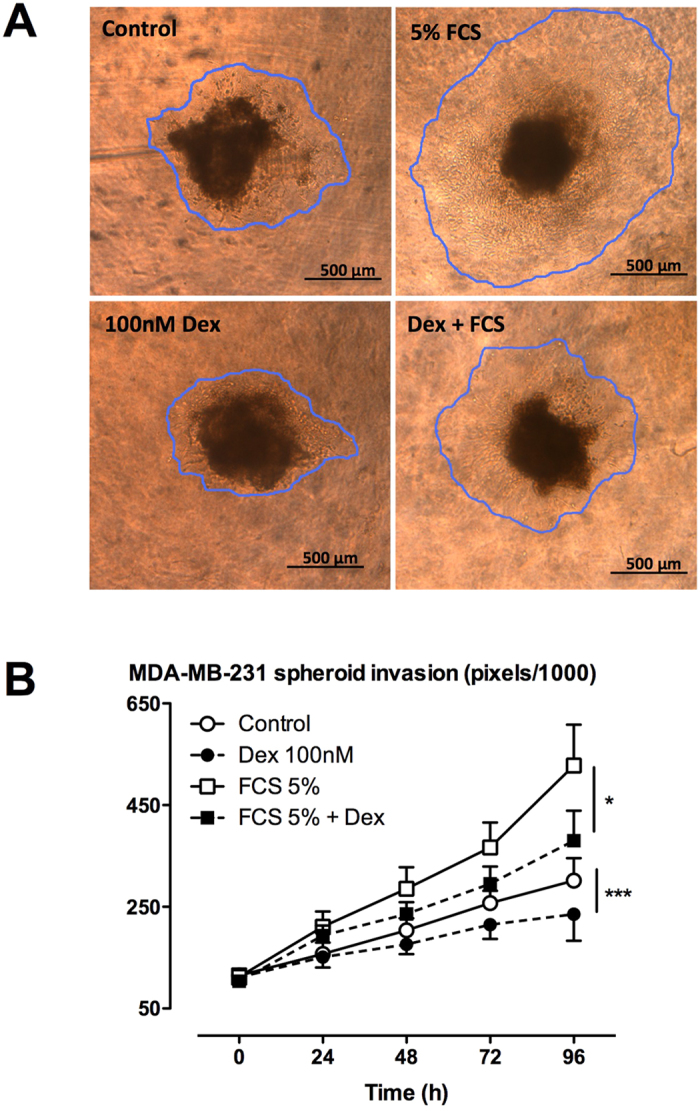
Effect of glucocorticoids on collagen matrix invasion of 3-dimensional MDA-MB-231 cell spheroids. 3-dimensional MDA-MB-231 cell spheroids were generated in low-adhesion round bottom 96 well plates then embedded into type-I fibrillar collagen derived from rat-tails (450 μg/mL). Collagen-embedded spheroids were then treated with dexamethasone (100 nM) or vehicle control (0.01% DMSO) for 30 min prior to the addition of fetal calf serum (FCS, 5% v/v). Static images were taken every 24 hours for 96 h total using an Olympus IX51 microscope (**A**). The cell-containing area was traced using ImageJ software to determine the extent of invasion (**B**). Data are expressed as the area of invasion in pixels/1000 and presented as mean ± SEM from n = 8 independent experiments. *P < 0.05, ***P < 0.001 from two-way ANOVA with Bonferroni post-hoc test.

Dexamethasone inhibited both chemotactic and chemokinetic migration (induced by 1% FCS) in the modified Boyden chamber assay (Fig. 1E). The chemotactic and chemokinetic migratory effects of FCS were enhanced when the Transwell inserts were coated with Matrigel™ (Fig. 1F). However, this enhanced migration remained sensitive to attenuation by dexamethasone (Fig. 1F). Dexamethasone also slowed the basal and FCS-stimulated matrix invasion of MDA-MB-231 cells in a 3-dimensional cell-spheroid based assay (Fig. 2).

### Glucocorticoid inhibition of MDA-MB-231 cell migration is sensitive to the GR antagonist RU486 and the protein synthesis inhibitor cycloheximide

**Figure 3:**
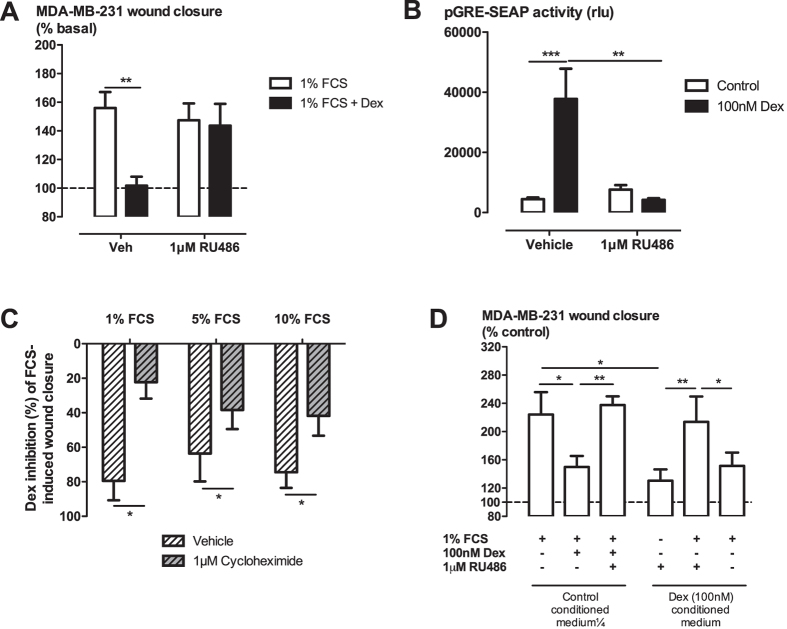
Glucocorticoid inhibition of MDA-MB-231 cell migration is sensitive to the GR antagonist RU486 and the protein synthesis inhibitor cycloheximide, but does not involve a secreted mediator. Glucocorticoid receptor antagonist RU486 prevents dexamethasone (10 nM) inhibition of wound closure (**A**) at a concentration that fully inhibits pGRE-SEAP reporter construct activation (**B**). The extent of wound closure 15 h after FCS addition is expressed as a percentage of basal (**A**) and the extent of pGRE-SEAP activation is expressed as relative light units (RLU) from a chemiluminescence SEAP detection kit (**B**). The protein synthesis inhibitor cycloheximide also attenuates dexamethasone inhibition of cell migration (**C**). To account for differences in extent of migration from different FCS concentrations, results are expressed as percentage inhibition of wound closure (**C**). Dexamethasone-conditioned medium (4 h exposure) in which the glucocorticoid activity is prevented by RU486 does not influence cell migration in scrape wound healing assay (**D**). All data are presented as mean ± SEM of n = 3 independent experiments. *P < 0.05, **P < 0.01, ***P < 0.001 from two-way ANOVA with Bonferroni post-hoc test.

The GR transactivation antagonist and transrepression partial agonist RU486 (1 μM) had no effect on FCS-induced cell migration, but completely prevented the inhibition caused by dexamethasone (Fig. 3A). This concentration of RU486 was sufficient to fully inhibit GRE transactivation by dexamethasone in a reporter assay system (Fig. 3B).

In order to determine whether *de novo* protein synthesis was required for the inhibition of cell migration, cells were incubated with cycloheximide for 1 h prior to dexamethasone addition. Due to the capacity of cycloheximide to influence cell migration *per se*, higher concentrations of FCS were employed to generate a positive migration stimulus (Supplementary Figure S3). The extent of dexamethasone-induced inhibition of cell migration was decreased in the presence of cycloheximide over all the FCS concentrations used (Fig. 3C).

### Glucocorticoid inhibition of MDA-MB-231 cell migration is not mediated by a secreted product

Given cycloheximide attenuated the glucocorticoid inhibition of migration, the synthesis of new protein products is required to manifest the anti-migratory effect. We therefore next investigated whether a newly formed anti-migratory protein may be released from glucocorticoid-treated cells and act in an autocrine or paracrine manner to inhibit cell migration. As expected, conditioned medium from dexamethasone-treated cells inhibited FCS-induced migration of naïve cells (Fig. 3D). However, when RU486 was added to block the activity of the dexamethasone in the conditioned-medium, the FCS-induced migration was not influenced by the conditioned medium (Fig. 3D). These results suggest no stable anti-migratory mediator is released by MDA-MB-231 cells in response to 4 hours of dexamethasone treatment.

### MDA-MB-231HM.LNm5 breast cancer cells are spontaneously metastatic to lymph node, liver and lung in immuno-compromised mice

The metastatic capacity of MDA-MB-231HM.LNm5 cells was compared with that of parental MDA-MB-231 cells in immuno-compromised NSG mice using *in vivo* and *ex vivo* imaging. MDA-MB-231HM.LNm5 cells spontaneously metastasised to liver, lung and axillary lymph nodes in all three mice inoculated (Supplementary Figure S4, Table S1). In addition, spleen metastases were also observed in one mouse while large paraspinal metastases between the kidneys were evident in two animals. Notably, the extent of metastasis to liver, lung, and lymph node appeared independent of local tumour recurrence following surgical resection of the primary tumour. Distant metastasis was not evident in mice inoculated with parental MDA-MB-231 cells (data not shown).

### MDA-MB-231-HM.LNm5 cells show decreased cell migration and decreased glucocorticoid sensitivity compared to parental MDA-MB-231 cells

**Figure 4:**
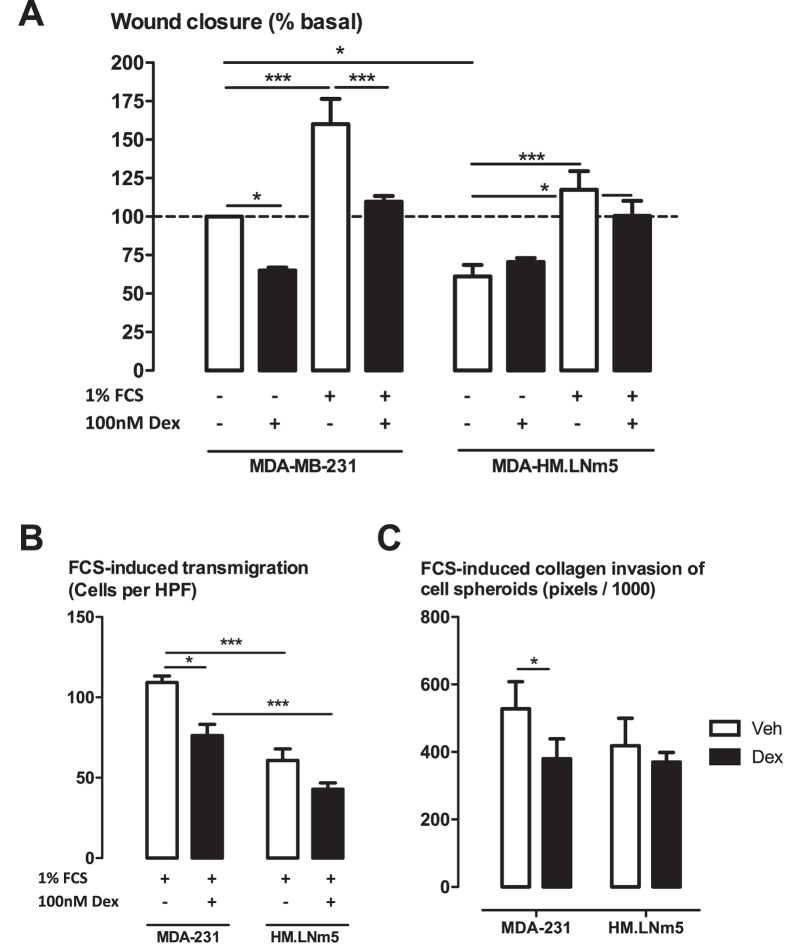
Effect of dexamethasone on migration of the highly metastatic MDA-MB-231-HM.LNm5 cell line. Migration of parental MDA-MB-231 and highly metastatic MDA-MB-231-HM.LNm5 cell line derived from lymph node metastasis of MDA-MB-231 cells were compared in scrape wound healing assay (**A**), modified Boyden chamber assay (**B**) and spheroid matrix invasion assay (**C**). Dexamethasone (100 nM) was added for 30 min prior to stimulation with 1% v/v FCS (**A**,**B**) or 5% v/v FCS (**C**). Data are presented as mean ± SEM for n = 3 (**A**), n = 3–7 (**B**), n = 4 (**C**) independent experiments. *P < 0.05, ***P < 0.001 from two-way ANOVA with Bonferroni post-hoc test.

The highly metastatic MDA-MB-231-HM.LNm5 cell line showed reduced basal migration in the scrape wound healing assay compared to the parental line (Fig. 4A). FCS (1%) enhanced wound closure in both the highly metastatic MDA-MB-231-HM.LNm5 cell line and in the parental cell line. Remarkably, dexamethasone influenced neither basal migration, nor FCS-induced migration in the highly metastatic variant (Fig. 4A). Similar observations were made in the Boyden chamber assay, where the highly metastatic variant showed reduced FCS-induced migration and no effect of dexamethasone treatment (Fig. 4B). In the 3D spheroid invasion assay, the highly metastatic variant cell line showed similar levels of collagen invasion to the parental cell line (Fig. 4C). However, no effect of dexamethasone on MDA-MB-231-HM.LNm5 collagen invasion was observed (Fig. 4C).

### MDA-MB-231-HM.LNm5 cells show decreased glucocorticoid receptor expression and decreased glucocorticoid-responsive gene expression compared to parental MDA-MB-231 cells

**Figure 5:**
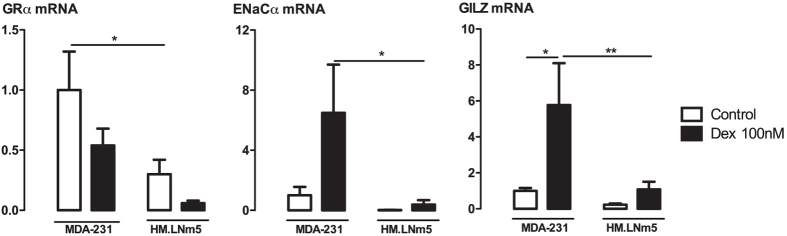
Glucocorticoid receptor and glucocorticoid-inducible gene expression in highly metastatic MDA-MB-231-HM.LNm5 cell line compared with parental MDA-MB-231 cells. qPCR was performed on cDNA derived from highly metastatic MDA-MB-231-HM.LNm5 cells and parental MDA-MB-231 cells treated with dexamethasone for 24 h. Target gene expression of glucocorticoid receptor (GRα; gene name: NR3C1), epithelial sodium channel alpha subunit (ENaCα; gene name: SCNN1A), glucocorticoid inducible leucine zipper (GILZ; gene name: TSC22D3) was normalised to 18 S rRNA housekeeping gene and is expressed as fold-change from levels in parental MDA control cells. Data presented as mean ± SEM from n = 4 independent experiments. *P < 0.05, **P < 0.01 from two-way ANOVA with Bonferroni post-hoc test.

Upon examination by qPCR, GR expression was significantly lower in MDA-MB-231-HM.LNm5 cells compared to parental cells (Fig. 5). There was also reduced expression of the dexamethasone-responsive genes encoding glucocorticoid-induced leucine zipper (GILZ, gene name TSC22D3) and epithelial sodium channel 1 alpha (ENaCα, gene name SCNN1A) (Fig. 5).

### Gene Ontology enrichment analysis identifies the most highly enriched gene ontologies relate to cell motility and migration in dexamethasone-treated MDA-MB-231 cells compared to MDA-MB-231-HM.LNm5 cells

Transcriptomic analysis of the parental and MDA-MB-231-HM.LNm5 cells detected 10352 genes above threshold levels of 10 cpm. A hierarchical list of differentially expressed genes between the Dex-treated MDA-MB-231 and MDA-MB-231-HM.LNm5 cell lines were subject to Gene Ontology (GO) enrichment analysis and 9703 genes were found to be associated with a GO term. Dex-treated parental MDA-MB-231 cells were found to have 199 enriched ontologies (p < 0.001) compared to Dex-treated MDA-MB-231-HM.LNm5 cells (Supplementary Figure S5A). Of these, the 8 most enriched ontologies (p < 10^−10^) all relate to locomotion and migration (Table 1, Supplementary Figure S5B).

### MDA-MB-231-HM.LNm5 cell transcriptome shows decreased dexamethasone-induced changes in migratory genes compared to parental MDA-MB-231 cell transcriptome

Further transcriptomic analysis was performed on dexamethasone-induced changes in migratory genes by examining the magnitude of change of Dex over control for genes directly annotated with GO terms positive regulation of cell migration” (GO:0030335) and “negative regulation of cell migration” (GO:0030336).

**Figure 6:**
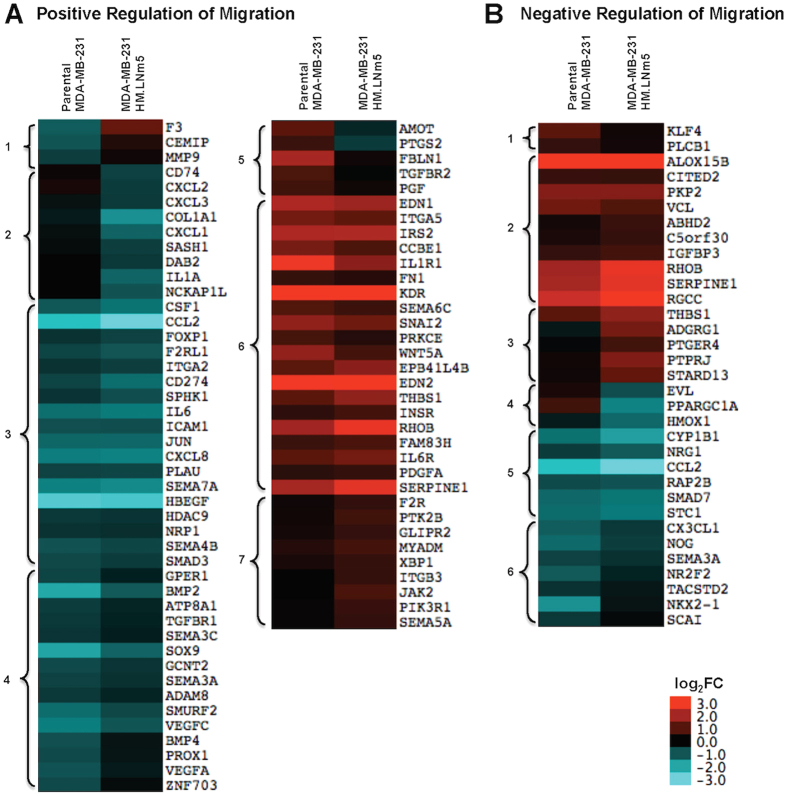
Dexamethasone-induced changes in migratory genes in the transcriptomes of MDA-MB-231-HM.LNm5 and parental MDA-MB-231 cell lines. RNA-Seq analysis was performed on dexamethasone-treated and control MDA-MB-231-HM.LNm5 and parental MDA-MB-231 cell lines. Dex-induced changes in expression of genes annotated with (**A**) GO:0030335 ‘positive regulation of cell migration’ or (**B**) GO:0030336 ‘negative regulation of cell migration’ in the amiGO Gene Ontology (GO) database were calculated as log_2_(fold change, FC, of Dex/Control) for each cell line. Genes with low read count (<10 cpm in all samples) or low Dex regulation (<1.5 fold) were excluded from the analysis. The remaining genes were hierarchically clustered using an uncentered correlation as distance measure and an average linkage using Cluster 3.0 software and are presented as heat maps generated by java TreeView. Clusters of genes showing a similar pattern of Dex-regulation have been numbered 1–7 for GO:0030335 and 1–6 for GO:0030336.

Parental and MDA-MB-231-HM.LNm5 cells revealed the detectable expression of 233 genes annotated with the term “positive regulation of cell migration” (GO:0030335). Of these 233 genes, 78 genes were regulated by dexamethasone in either cell line (Fig. 6A). Of the 36 genes annotated with the term “positive regulation of cell migration” that were repressed to less than 0.66 fold by dexamethasone in the parental cell line (Fig. 6A, cluster 1, 3, 4), 15 were repressed to a lesser extent (Fig. 6A, cluster 4) and 3 were stimulated (Fig. 6A, cluster 1) in the MDA-MB-231-HM.LNm5 cells. Of the 24 genes annotated with the term “positive regulation of cell migration” which were stimulated by ≥1.5 fold by dexamethasone in the parental cell line (Fig. 6A, cluster 5 & 6), 9 were repressed to a lesser extent (Fig. 6A, cluster 6) and 5 were repressed (Fig. 6A, cluster 5) in the MDA-MB-231-HM.LNm5 cells. 9 genes (Fig. 6A, cluster 7) were only induced in the MDA-MB-231-HM.LNm5 cell line and not in the parental cell line.

128 genes annotated with the term “negative regulation of cell migration” (GO:0030336) were detected in the RNA-Seq analysis above threshold levels as specified in the methods section. Of these, 33 genes were regulated by dexamethasone in either the parental or MDA-MB-231-HM.LNm5 cell line (Fig. 6B). 2 genes were selectively induced in the parental line (Fig. 6B, cluster 1) and 5 genes were selectively induced in the MDA-MB-231-HM.LNm5 cell line (Fig. 6B, cluster 3). In contrast, 3 genes were selectively repressed in the parental line (Fig. 6B, cluster 4) and 7 genes were selectively repressed in the MDA-MB-231-HM.LNm5 cell line (Fig. 6B, cluster 6).

## Discussion and Conclusions

This work has shown for the first time that glucocorticoids inhibit 2- and 3-dimensional migration of the ER-negative MDA-MB-231 human breast cancer cell line. This study has added to the growing body of evidence that glucocorticoids inhibit cell migration^36^,^37^,^38^,^39^. We found similar inhibitory effects in ER-positive MCF-7 and ER-negative MDA-MB-231 breast tumour cells suggesting that the presence of the ER is not permissive for the anti-migratory effects of dexamethasone, despite a known connection between the ER and cellular migration^41^ and the fact that ER-negative tumours are often intrinsically more metastatic than ER-positive tumours^34^.

Given cellular migration is a critical component of metastasis, we also examined the changes in migratory phenotype and glucocorticoid sensitivity that occur during selection of cells with high metastatic potential. The highly metastatic MDA-MB-231-HM.LNm5 cell line showed decreased basal and FCS-induced migration and, surprisingly, glucocorticoid treatment of this cell line caused no inhibition of migration. We therefore conclude that selection for high metastatic potential has resulted in a less migratory cell phenotype and a decreased responsiveness to the anti-migratory effect of glucocorticoids.

The observation that the highly metastatic cell line has decreased migratory phenotype is unexpected although not entirely unprecedented, as inverse relationships between migratory behaviour and metastasis have been previously observed in melanoma^42^. This finding highlights the complex nature of metastasis whereby cells do not merely acquire a pro-migratory and pro-invasive phenotype but also become highly proliferative, evade apoptotic mechanisms, become insensitive to anti-growth signals, promote sustained angiogenesis and develop a limitless replicating potential^43^. Our results might suggest that the metastatic selection was driven by a change in one or more of these other mechanisms, and the resulting effect on migration was secondary to these changes. Alternatively the decrease in migratory phenotype may have promoted the metastatic phenotype by allowing cells more time to adhere and grow at metastatic sites, or may have facilitated evasion of immune recognition mechanisms.

Interestingly, the sensitivity of MDA-MB-231 cells to glucocorticoid treatment was altered by the selection for metastatic potential. MDA-MB-231-HM.LNm5 cells showed no change in cell migration or invasion upon treatment with dexamethasone. We also found that the MDA-MB-231-HM.LNm5 cell line expresses much lower levels of GRalpha mRNA and this correlated with reduced dexamethasone-induction of glucocorticoid-responsive genes. The emergent resistance to the actions of glucocorticoids through metastatic selection is to our knowledge a novel finding. Importantly, it was not a result of selection pressure following drug administration, since the *in vivo* cycling method^40^,^44^ used to generate the highly metastatic cell line did not expose the cells to high levels of glucocorticoid, but rather the resistance occurs against a physiological background concentration of corticosterone. Our data also suggest that the decrease in GRalpha and concomitant decrease in glucocorticoid sensitivity may be permissive to the establishment of the more metastatic phenotype. For example, glucocorticoids are well known to be anti-angiogenic^45^, through both direct effects on endothelial cells^46^, as well as through suppression of angiogenic factor release from cells neighbouring the vasculature, including tumour cells^47^. It is therefore intriguing to speculate that a reduction in glucocorticoid sensitivity has promoted the release of pro-angiogenic mediators from the MDA-MB-231-HM.LNm5 cell line, causing an increase in metastatic potential through an increase in angiogenesis.

Several steps in this study have been taken to ensure that migratory responses rather than cellular proliferation were assessed. Several distinct assays of migration assessed: movement of tumour cells as 2-dimensional cellular monolayers; individual cell migration through transwell pores in the presence and absence of extracellular matrix; as well as invasion in 3-dimensions through extracellular matrix. These separate approaches allowed us to increase our confidence in our conclusions. Each experiment was carefully designed to use minimal FCS concentrations to stimulate migration and we have kept the duration of experiments as short as possible in order to minimise the contribution of proliferation. Despite these precautions, it was still necessary to directly assess the influence of dexamethasone exposure on MDA-MB-231 cell proliferation. As expected, FCS stimulation caused an increase in proliferation as measured by both cell enumeration and Resazurin assay. However, dexamethasone exposure had no effect on basal or FCS-stimulated cell number in either assay. We therefore interpret the glucocorticoid influences on cell migration as direct rather than secondary to changes in cell number.

The inhibition of migration by glucocorticoids in the parental MDA-MB-231 cell line was completely prevented by the GR antagonist RU486, suggesting that this effect is mediated through canonical GR transactivation signalling mechanisms^48^. The glucocorticoid effect required production of new protein products, as evidenced by reduced inhibition in the presence of cycloheximide. This observation ruled out non-genomic mechanisms of glucocorticoid actions, such as modulation of PI3K and JNK signalling pathways^18^,^20^. However, there was no evidence of mediation by a secreted product. Further work will be required to determine the precise mechanism through which GR transactivation leads to inhibition of migration. However, this could be difficult to establish due to the multitude of inter-dependent GR mechanisms^20^,^21^,^49^. Interestingly, we found distinct transcriptomic patterns between the highly metastatic and parental cell lines following dexamethasone treatment in genes controlling both positive and negative regulation of migration. This suggests that selection for metastatic potential has resulted in altered glucocorticoid signalling mechanisms and an impaired ability to activate pathways leading to inhibition of migration. Interestingly, different glucocorticoids have recently been shown to differentially activate the transcription of different genes products^50^ foreshadowing future drug optimisation strategies for improved efficacy^49^ in attenuating cell migration. Importantly, since the discovery of the anti-emetic effect of glucocorticoids in the early 1980 s, there has been little or no optimisation for this indication. It remains likely that certain glucocorticoid agonists have better efficacy/safety profiles than those currently used (dexamethasone or methylprednisolone).

In this study, we have presented evidence that the sensitivity of MDA-MB-231 breast tumour cells to dexamethasone is dependent on the stage of metastatic progression. We do not yet know what molecular alterations have occurred during the phenotype change from parental MDA-MB-231 cell line to the highly metastatic MDA-MB-231-HM.LNm5 cell line. Nor do we know at what stage of metastatic phenotype selection the resistance to glucocorticoid inhibition emerged. In future work it will also be important to establish whether other similar sublines express similar characteristics following metastatic selection since the current study is limited to one cell line. Importantly, the emergence of glucocorticoid resistance during metastatic selection may go some way to explaining the apparent disparity between clinical evidence that activation of the GR is associated with a worse prognosis in estrogen receptor (ER)-negative breast cancer^32^,^33^, and *in vitro* studies showing inhibitory actions of glucocorticoids in commonly used cell lines. These findings highlight the highly plastic nature of tumour cells during metastasis, and underscore the need to more fully understand the direct effect of adjuvant glucocorticoid treatment on cancer cell biology at different stages of progression.

## Methods

### Cell Culture

MDA-MB-231 and MCF-7 breast tumour cells (ATCC, Manassas, VA, USA) were cultured in phenol-red free RPMI 1640 and DMEM respectively supplemented with 10% (v/v) heat-inactivated fetal bovine serum (FCS), 2 mM L-glutamine, 1 mM nonessential amino acids, 1 mM sodium pyruvate, 15 mM HEPES solution, 100 U/mL penicillin and 100 μg/mL streptomycin as previously described^51^,^52^. The highly metastatic MDA-MB-231-HM.LNm5 cell line was generated through a two-step process whereby a cell population initially selected by 6 cycles of transplanting spontaneous MDA-MB-231 pulmonary metastases to the mammary fat pad^44^ (designated MDA-MB-231-HM and kindly provided by ZM Shao and ZL Ou (Breast Cancer Institute, Fudan University, Shanghai, China) were inoculated into the mammary fat pad of a female BALB/c-SCID mouse, following which the axillary lymph node metastases were isolated. All experiments involving mice had approval from the Peter MacCallum Animal Experimentation Ethics Committee and were carried out in accordance with the Australian code for the care and use of animals for scientific purposes (8^th^ edition, 2013). MDA-MB-231-HM.LNm5 cells were cultured in RPMI 1640 supplemented as above.

### Assessment of metastatic potential

The metastatic capacity of MDA-MB-231HM.LNm5 cells was compared with that of parental MDA-MB-231 cells in immuno-compromised NOD scid gamma (NSG) mice. To facilitate optical imaging of tumours *in vivo*, both parental MDA-MB-231 and MDA-MB-231HM cells were transduced with retrovirus encoding tdTomato fluorescent protein, selected with Blasticidin S and bulk sorted for tdTomato expression by flow cytometry (FACSAria, Beckton Dickinson) as previously described^53^. Both populations were also transduced with retrovirus encoding Firefly luciferase and selected using puromycin^53^. Cells (n = 3 for each line) were inoculated into the 4^th^ inguinal mammary gland of NSG mice and tumours were allowed to form (one tumour per mouse). Primary mammary tumours were surgically resected 22 days after inoculation. *In vivo* bioluminescence imaging was performed 13 days after primary tumour resection to detect luciferase-positive proximal metastatic lesions. Mice were culled 21 days following resection and distant metastases were evaluated by *ex vivo* imaging of tdTomato fluorescence in whole organs (lung, liver, spine, lymph node and spleen) using fluorescent stereomicroscopy.

### Scrape wound healing assay

MDA-MB-231 cells were subcultured in 24-well plates at a density of 10^5^ cells/cm^2^ and grown in serum-containing media until confluency was reached before being starved for 24 hours in serum-free media containing 0.25% BSA. A sterile 200 μL pipette tip was used to scrape away a central portion of cells and media was replaced to remove dislodged cells and debris. Cells were treated with glucocorticoids (1–100 nM dexamethasone, 1 μM hydrocortisone, 100 nM methylprednisolone, 100 nM budesonide) for 30 minutes prior to stimulation with FCS (0.1–5%). The concentrations of glucocorticoids used are able to fully saturate the glucocorticoid receptor and show maximal effects in a wide variety of cell types including airway smooth muscle cells^54^, airway epithelial cells^50^, breast cancer cells^55^, peripheral blood mononuclear cells^56^. In selected experiments, GR antagonist 1 μM RU486 was added 30 min prior to glucocorticoids. Protein synthesis inhibitor cycloheximide (1 μM) was added 1 h prior to glucocorticoids. Conditioned media was generated by exposing confluent, serum-starved MDA-MB-231 cells to dexamethasone or vehicle for 4 hours before media was transferred to a denuded 24-well plate and treatments added as above. Cells were imaged every 30 minutes for 15 hours using the Leica DMI6000B live cell imaging microscope, being maintained at 37 °C in an atmosphere of room air containing 5% CO_2_. The optical density (grey value) of the denuded region was determined by manual delineation of the wound area using ImageJ (version 1.46). This value was subtracted from the grey values of the same area in subsequent frames. In individual experiments, the wound closure at 15 hours in untreated (control) culture was used to normalise all other values.

### Boyden chamber assay

Chemotactic and chemokinetic migration was observed using polycarbonate cell culture inserts containing 8 μm pores (Costar #3422). Some inserts were coated with 7 μL Matrigel™ (BD Biosciences, North Ryde, NSW, Australia), diluted 1:3 with serum-free RPMI to assess cell invasion. Inserts were centrifuged for 10 minutes at 270 *g* to generate an even coating. MDA-MB-231 cells grown to confluence in 25 cm^2^ flasks were serum-starved for 24 h then dislodged using trypsin-EDTA. 50,000 cells in 150 μl serum-free RPMI were seeded into the upper compartment of cell culture inserts and left to adhere for 6 h with 500 μl serum-free RPMI in the bottom compartment. 100 nM dexamethasone was added to both compartments 30 min before FCS (1% v/v) was added to stimulate chemokinetic migration (FCS added to both compartments) or chemotactic migration (FCS added to bottom compartment only). Inserts were incubated for 20 hours at 37 °C in 5% CO_2_ at which time non-migrated cells were scraped from the upper side of the polycarbonate membrane and migrated cells on the underside of the membrane were stained using Diff-Quick™ (Thermo Fisher, Scoresby, VIC, Australia). Briefly, inserts were washed twice with PBS, fixed with DiffQuick™ fix for 2 min then stained in DiffQuick™ red stain for 10 seconds, followed by DiffQuick™ blue for 6 seconds, and then washed twice in PBS. Membranes were excised from inserts using a scalpel, placed onto microscope slides to air dry and coverslipped using DEPEX mountant. Cells were visualised by light microscopy at a magnification of 400×. The number of cells was counted, with five central, adjacent fields of view averaged for each insert.

### 3D spheroid matrix invasion assay

Round bottom 96-well plates (Corning, Tewksbury, MA, USA) were coated with 0.5% poly(2-hydroxyethyl methacrylate) (poly-HEMA) to minimise cell adhesion. Cell spheroids were generated in 96 h from 5,000 cells which were seeded into each poly-HEMA coated well in a non-gel forming concentration (2.5%) of Cultrex^®^ extracellular matrix (R&D Systems, Minneapolis, USA) in RPMI medium.

Spheroid matrix invasion was assessed by embedding cell spheroids in type-I fibrillar collagen derived from rat-tails (450 μg/mL). The spheroid-containing collagen was incubated at 37 °C for 1 hour to allow the collagen to set before 100 μL serum-free RPMI was added along with dexamethasone (100 nM) or vehicle (0.01% DMSO) for 30 minutes prior to the addition of FCS (5%). Static images of the spheroids were taken every 24 hours using an Olympus IX51 microscope. The cell-containing area was traced using ImageJ software to determine the extent of invasion.

### pGRE-SEAP reporter assay

MDA-MB-231 cells were transiently transfected with a pGRE-SEAP reporter construct (Clontech, Mountain View, CA, USA) as previously described^57^. Briefly, cells were transfected for 6 hours with a pGRE-SEAP reporter construct (150 ng/well) that was complexed with Lipofectamine 2000 reagent (1 μL/well) in 100 μL/well of OptiMEM for 20 min at room temperature prior to addition to cells. The day after transfection, cells were treated with RU486 (1 μM) for 30 min prior to glucocorticoid stimulation. After 24 h, supernatants were collected and the amount of secreted human placental alkaline phosphatase (SEAP) was determined using a chemiluminescence kit (Roche Applied Science, NSW, Australia).

### RNA extraction and RT-qPCR

MDA-MB-231 and MDA-MB-231-HM.LNm5 cells were subcultured into 24-well plates at 10^5^ cells/cm^2^ and grown for 48 hours before being serum-starved for 24 h. Cells were treated with dexamethasone (100 nM) for 24 hours before total RNA was harvested using TRIzol^®^ (Life technologies). Reverse transcription was carried out using the High Capacity cDNA Reverse Transcription Kit (Invitrogen, Mulgrave, VIC, Australia). Reactions of 5 μL total volume were performed using 2 μl total RNA (containing 100 ng RNA), 2.5 μL 2× RT Buffer, 0.25 μL 20× RT Enzyme Mix and 0.25 μL DEPC-treated water. Thermal conditions for the reaction were 37 °C, 60 minutes; 95 °C, 5 minutes which was performed on a Mastercycler^®^ Pro (Eppendorf, Hamburg, Germany). The resulting cDNA was diluted with 145 μL DEPC-treated water and stored at −20 °C. Real-time PCR was performed in triplicate for each gene of interest in a 384-well plate using ABI Prism 7900HT sequence detection system (Applied Biosystems). Each 5 μL reaction consisted of 1.5 μL diluted cDNA, 2.5 μL iTaq™ universal SYBR^^®^^ Green Supermix (Bio Rad, Gladesville, NSW, Australia) and 0.5 μL of each of the relevant forward and reverse primers (Table 2). The conditions for PCR amplification were: 50 °C for 2 min then 95 °C for 10 min followed by 40 cycles of 95 °C for 0.15 min, and 60 °C for 1 min. The threshold cycle determined for each gene was normalized against that obtained for 18S ribosomal RNA, which was measured as internal control.

### Cell enumeration

MDA-MB-231 cells were subcultured in 24-well plates at a density of 50,000 cells/cm^2^ and grown in serum-containing media for 24 hours before being starved for 24 hours in serum-free media containing 0.25% BSA. Cells were treated with 100 nM dexamethasone for 30 min prior to stimulation with FCS 5% for 48 hours. Cells were then exposed to 0.12% (w/v) trypsin for 5 minutes before being neutralised in a 0.067% Trypan blue/FCS solution. The number of cells per well was then manually counted using a haemocytometer in duplicate.

### Resazurin reduction assay

Reduction of the redox dye resazurin to resorufin was used as a measure of cellular proliferation^58^. MDA-MB-231 cells were seeded in 96 well flat-bottomed plates at 36,000 cells/cm^2^ and allowed to adhere/proliferate for 24 hours before being starved for 24 hours in serum-free RPMI. Cells were pre-incubated with 100 nM dexamethasone for 30 min before stimulation with 1–5% (v/v) FCS for 48 h. Medium was aspirated and cells were incubated with resazurin reagent containing 1.5% (w/v) Resazurin, 0.25 (w/v) methylene blue, 2.9% (w/v) potassium hexacyanoferrate (III) and 4.22% (w/v) potassium hexacyanoferrate (II) trihydrate for 2 h at 37˚C in 5% CO_2_. Resorufin formation was measured fluorometrically at excitation of 570 nm and emission of 620 nm using a FlexStationIII (Molecular Devices, Sunnyvale, CA, USA).

### Transcriptomic Analysis

MDA-MB-231 and MDA-MB-231-HM.LNm5 cells were serum-starved for 24 h then treated with dexamethasone (100 nM) or vehicle control for 24 hours after which total RNA was extracted using TRIzol^®^ (as above). RNA-Seq libraries were prepared from 500 ng total RNA using NEBNext Ultra RNA library prep kit for Illumina (#E7530) with NEBNext Poly(A) mRNA Magnetic Isolation Module (#E7490). Prior to library preparation, RNA was confirmed to be of high quality (RIN > 8) by Agilent Bioanalyzer 2100 analysis. Paired end 2 × 50 bp rapid sequencing was performed on an Illumina HiSeq 2500 in the Melbourne Translational Genomic Platform, University of Melbourne. Raw data was filtered by removing reads with adaptor sequences, reads where the percentage of unknown bases is greater than 10%, and reads considered low quality (where bases with quality value less than or equal to 5 constitute greater than 50% of base reads) to obtain clean reads. All subsequent analyses are based on clean reads only. Clean reads were mapped to the human reference genome GRCh38-hg38 assembly using Rsubread package^59^. Reads that were aligned to annotated coding regions of the genome were counted using featureCounts^60^ and gene expression values were calculated for each gene as counts per million (cpm). Genes that did not reach a threshold level of 10 cpm in at least one condition were excluded from further analysis. Gene Ontology (GO) enrichment analysis was performed using GOrilla^61^,^62^ (http://cbl-gorilla.cs.technion.ac.il) on a hierarchical list of differentially expressed genes between the Dex-treated MDA-MB-231 and MDA-MB-231-HM.LNm5 cell lines. Further GO annotation analysis was carried out on genes annotated with GO:0030335 ‘positive regulation of migration’ and GO:0030336 ‘negative regulation of migration’ according to the AmiGO 2 database (version 2.3.2). For this analysis, Dex-induced changes were calculated as log2(fold change, FC) of Dex over Control for each cell line. We then performed hierarchical clustering using an uncentred correlation as distance measure and an average linkage using Cluster 3.0 software (http://bonsai.ims.utokyo.ac.jp/~mdehoon/software/cluster) and visualized clustered Heat maps using java TreeView (http://jtreeview.sourceforge.net).

### Statistical analysis

Results are expressed at mean ± SEM from *n* independent experiments (performed on separate days on cells from a different passage) and analysed as grouped data. Scrape wound healing is expressed as a percentage of the total control migration observed over 15 hours. Modified Boyden chamber data are expressed as the number of migrated cells per high-powered field (average of 5 fields). pGRE-SEAP activity data are expressed as relative light units. Quantitative PCR data are expressed as the fold change in expression from control. Cell viability data are expressed as a percentage of the number of control cells counted. One-way or Two-way ANOVA with repeated measures was performed using GraphPad Prism 5.0 on non-normalised data (GraphPad, San Diego, CA, USA). *Post hoc* Dunnett’s and Bonferroni tests were then performed to ascertain significance. P < 0.05 was considered to be statistically significant.

## Additional Information

**How to cite this article:** Fietz, E. R. *et al*. Glucocorticoid resistance of migration and gene expression in a daughter MDA-MB-231 breast tumour cell line selected for high metastatic potential. *Sci. Rep.* **7**, 43774; doi: 10.1038/srep43774 (2017).

**Publisher's note:** Springer Nature remains neutral with regard to jurisdictional claims in published maps and institutional affiliations.

## Supplementary Material

Supplementary Information

## Figures and Tables

**Table 1:** Highly enriched gene ontologies ascertained through GOrilla analysis of RNA-seq experiment comparing Dexamethasone treated MDA-MB-231 and MDA-MB-231-HM.LNm5 cells.

GO term	Description	P-value	FDR q-value	Fold enrichment
GO:0051272	positive regulation of cellular component movement	2.62 × 10^−11^	3.24 × 10^−7^	3.16
GO:0040017	positive regulation of locomotion	3.25 × 10^−11^	2.01 × 10^−7^	3.09
GO:2000147	positive regulation of cell motility	4.03 × 10^−11^	1.67 × 10^−7^	3.18
GO:0040012	regulation of locomotion	2.18 × 10^−10^	6.77 × 10^−7^	2.79
GO:0030334	regulation of cell migration	2.27 × 10^−10^	5.64 × 10^−7^	2.54
GO:0051270	regulation of cellular component movement	2.46 × 10^−10^	5.08 × 10^−7^	2.90
GO:2000145	regulation of cell motility	3.22 × 10^−10^	5.70 × 10^−7^	2.96
GO:0030335	positive regulation of cell migration	3.35 × 10^−10^	5.19 × 10^−7^	3.08

**Table 2:** Primer sequences for RT-PCR.

Gene	Forward Primer	Reverse Primer
r18s	CGC CGC TAG AGG TGA AAT	TCT TGG CAA ATG CTT TCG CTC
GILZ	TCC TGT CTG AGC CCT GAA GAG	AGC CAC TTA CAC CGC AGA AC
GR	TCA ACT GAC AAA ACT CTT GG	GCA ATA GTT AAG GAG ATT TTC AAC C
ENaCα	AGC ACA ACC GCA TGA AGA C	TGA GGT TGA TGT TGA GGC TG
