# Supplementary material for: Glucocorticoid resistance of migration and gene expression in a daughter MDA-MB-231 breast tumour cell line selected for high metastatic potential
Source: Sci Rep. 2017 Mar 6;7:43774. doi: 10.1038/srep43774 (PMC5338339; doi:10.1038/srep43774)
Supplement: Supplementary Information [file srep43774-s1.pdf]

SUPPLEMENTARY FIGURES

RESEARCH ARTICLE

Glucocorticoid resistance of migration and gene expression in a daughter MDA-MB-231 breast tumour cell line selected for high metastatic potential

Ebony R Fietz[1*], Christine R Keenan[1*], Guillermo Lopez Campos[2], Yan Tu[1], Cameron N Johnstone[3], Trudi Harris[1] and Alastair G Stewart[1]

*These authors contributed equally to the current work

[1]Department of Pharmacology and Therapeutics, University of Melbourne, Parkville, Victoria 3010, Australia
[2]Health and Biomedical Informatics Centre, University of Melbourne, Parkville, Victoria 3010, Australia
[3]Peter MacCallum Cancer Centre, East Melbourne, Victoria 3002, Australia

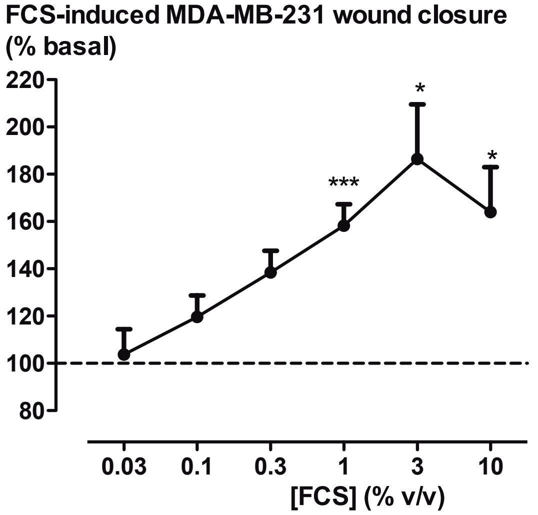

Supplementary Figure S1. Fetal calf serum (FCS)-induced MDA-MB-231 cell migration in scrape wound healing assay.
Wound infiltration was measured by the change in grey-value using ImageJ/FIJI software. The extent of wound closure 15 h after FCS addition is expressed as a percentage of basal and presented as mean ± SEM of n=4 independent experiments. *P<0.05, ***P<0.001 from repeated measures one-way ANOVA with Dunnett's post-hoc test.

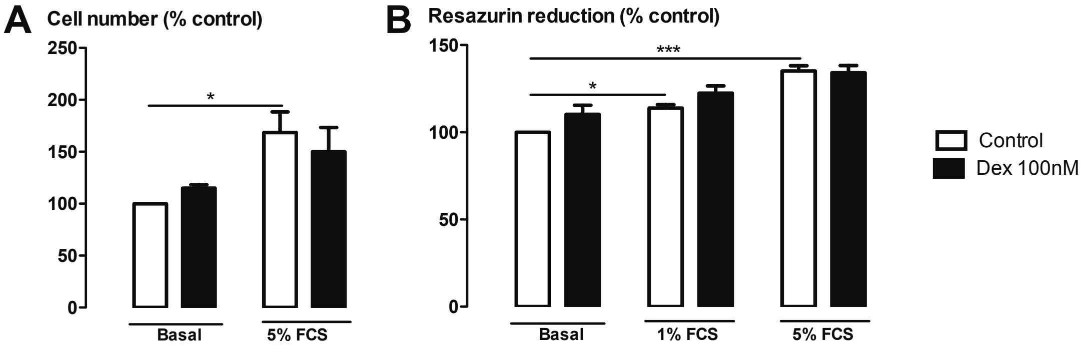

Supplementary Figure S2. Effect of dexamethasone on MDA-MB-231 cell proliferation.
Serum-starved MDA-MB-231 cells were pre-treated with dexamethasone (100nM) for 30 min then incubated with FCS (1-5% v/v) for 48 h. The level of proliferation was determined using cell enumeration by trypan blue (A) and by Resazurin assay (B). Results are expressed as a percentage of basal and is presented as mean ± SEM for n=3 independent experiments. *P<0.05, ***P<0.01 from two-way ANOVA with Bonferroni post-hoc test.

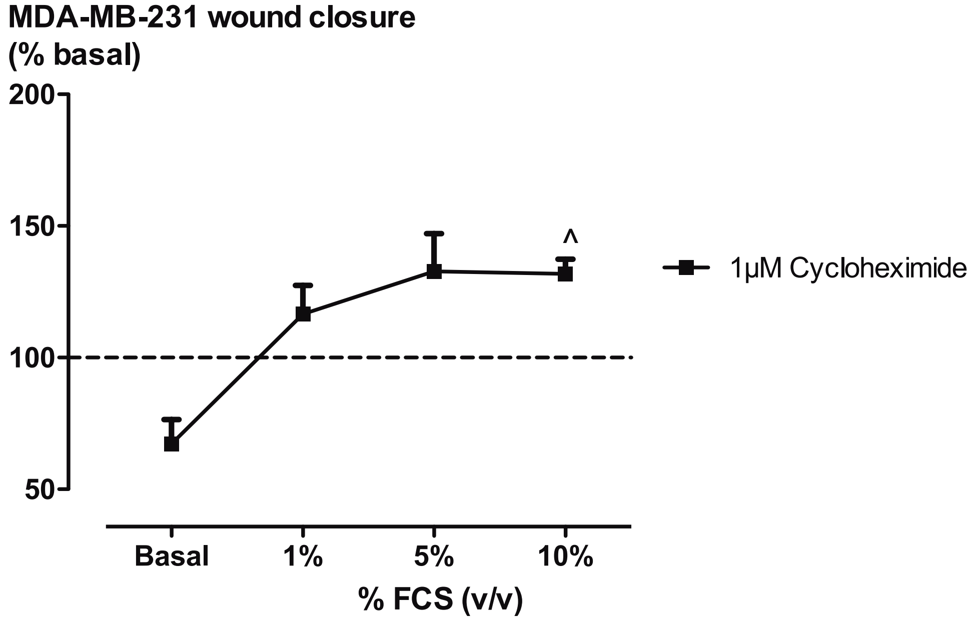

Supplementary Figure S3. Fetal calf serum (FCS)-induced MDA-MB-231 scrape wound healing closure in presence of cycloheximide

Wound infiltration was measured by the change in grey-value using ImageJ/FIJI software. The extent of wound closure 15 h after FCS addition is expressed as a percentage of basal and presented as mean ± SEM of n=4 independent experiments. ^P<0.05 from repeated measures one-way ANOVA with Dunnett's post-hoc test.

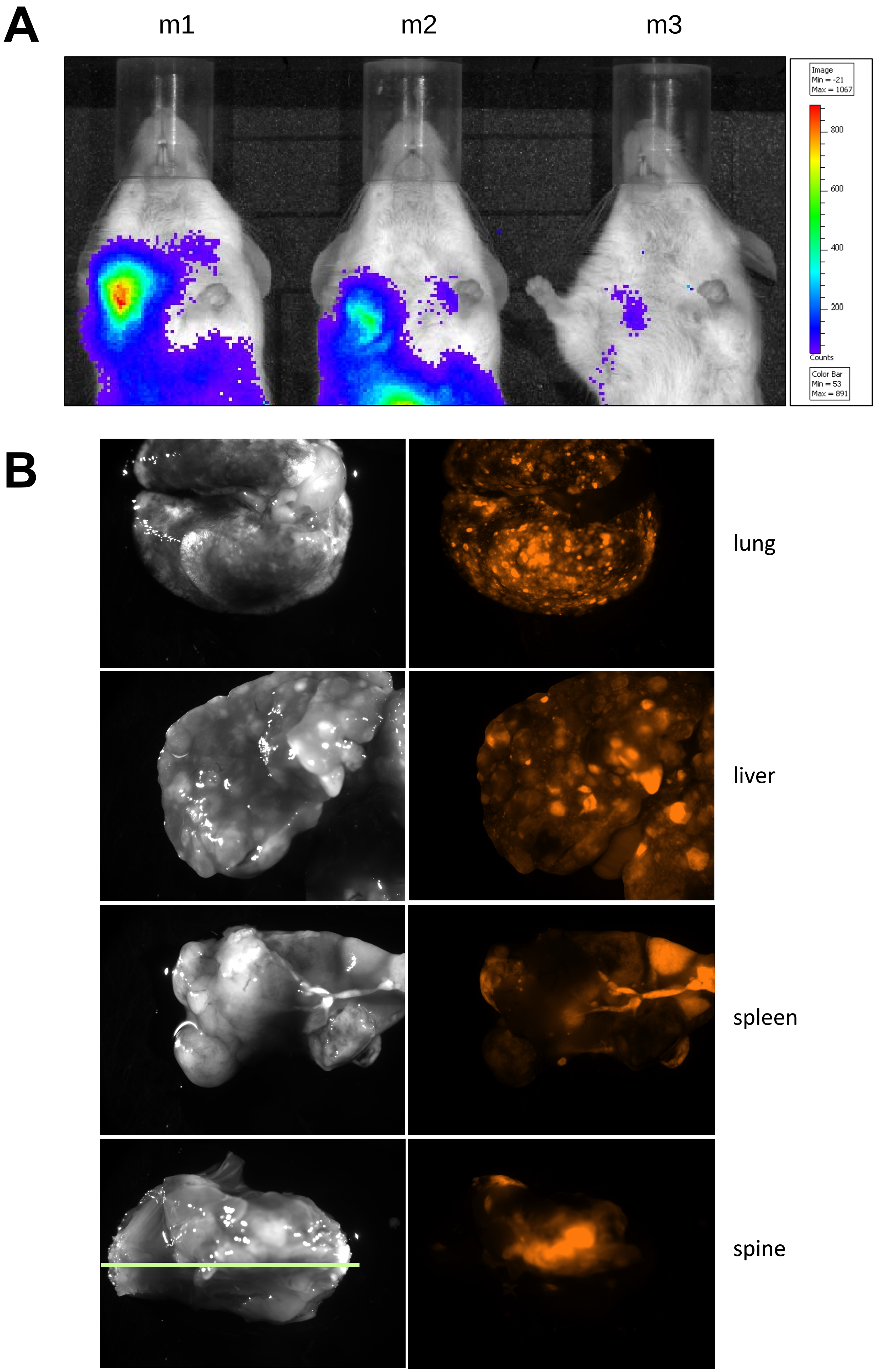
A
m1
m2
m3
Image
Min = -21
Max = 1067
800
600
400
200
Counts
Color Bar
Min = 53
Max = 891
B
lung
liver
spleen
spine

Supplementary Figure S4 (on previous page). ***In vivo*** **and** ***ex vivo*** **imaging of MDA-MB-231-HM.LNm5 cell metastatic lesions after primary tumour resection.** MDA-MB-231-HM.LNm5 cells were inoculated into the 4th inguinal mammary gland of NSG mice (n=3) and tumours were allowed to form (one tumour per mouse). Primary mammary tumours were surgically resected 22 days after inoculation. Mice were culled 21 days after resection. **A.** *In vivo* bioluminescence imaging of luciferase-positive proximal metastatic lesions formed 13 days after primary tumour resection (n=3). 30s exposure, medium bin. **B.** *Ex vivo* fluorescent imaging of distant organs from mouse 2 taken 21 days after primary tumour resection (x7 magnification). Left panels: greyscale. Right panels: fluorescence. Multiple metastases were evident in every lobe of the lung and liver. The green line indicates the approximate location of the spine.

Supplementary Table S1: Summary of spontaneous metastasis of MDA-MB-231HM.LNm5 primary tumours. The extent of local or distant recurrence was estimated using ex vivo imaging of tdTomato fluorescence in whole organs using fluorescent stereomicroscopy. Distant metastasis was not found in mice inoculated with parental MDA-MB-231. Legend: PT, primary tumour; LN, lymph node; m, mouse. X=weak, XXXX=strong.

Mouse	PT regrowth?	Lung mets?	Liver mets?	Paraspinal mets?	Axillary LN met?	Spleen mets?
m1	XXXX	XXXX	XXXX	Yes	Yes	No
m2	XX	XXXX	XXXX	Yes	Yes	Yes
m3	No	XXXX	XXXX	No	Yes	No

A

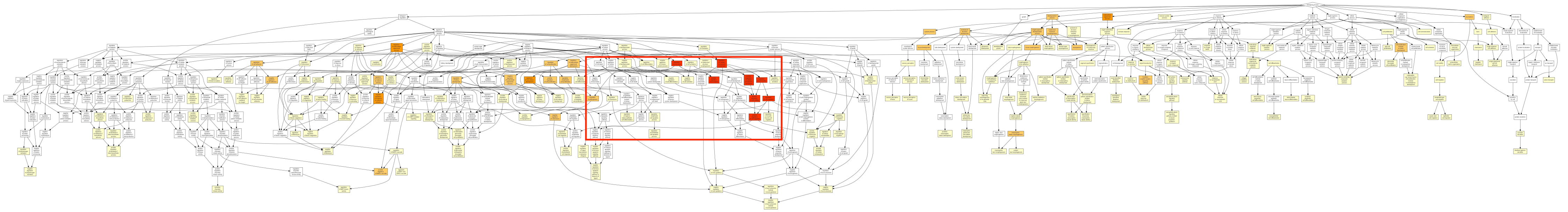

B

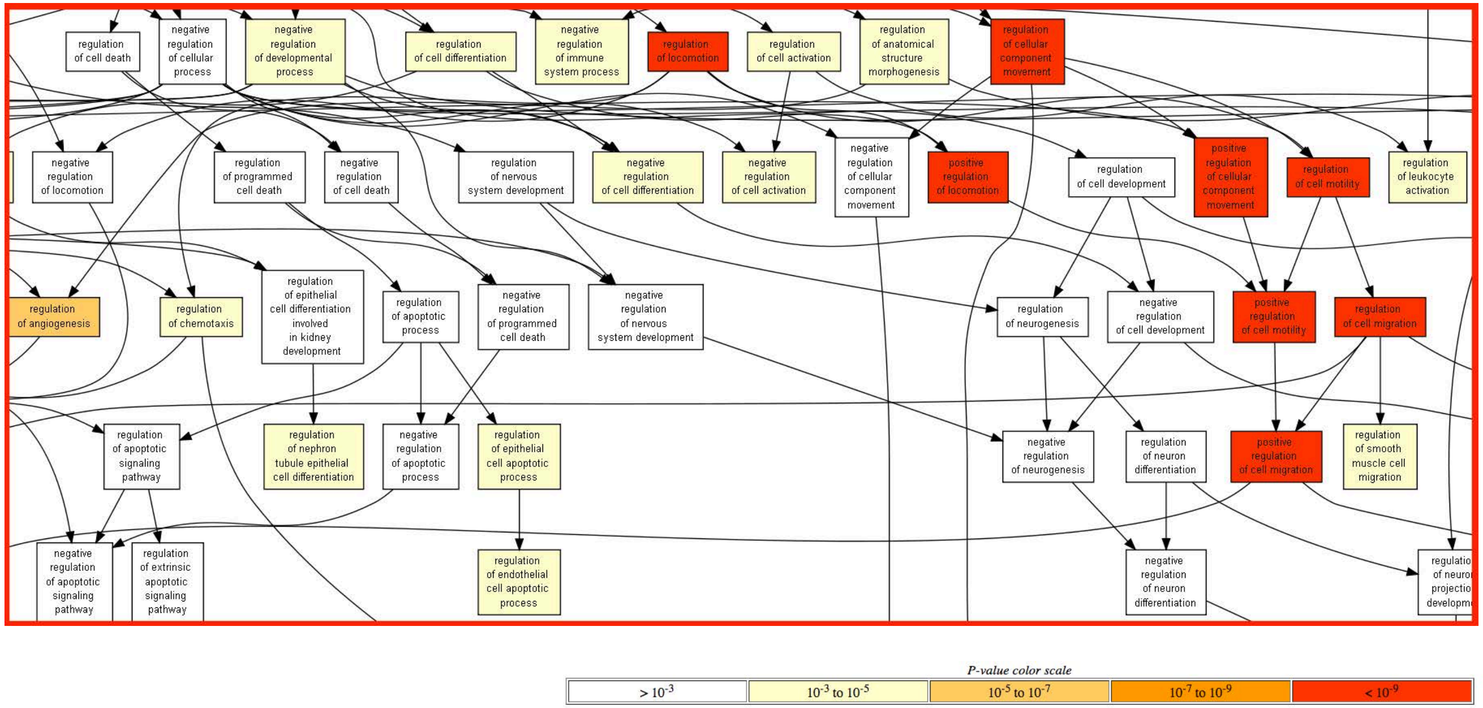
regulation of cell death
negative regulation of cellular process
negative regulation of developmental process
regulation of cell differentiation
negative regulation of immune system process
regulation of locomotion
regulation of cell activation
regulation of anatomical structure morphogenesis
regulation of cellular component movement
negative regulation of locomotion
regulation of programmed cell death
negative regulation of cell death
regulation of nervous system development
negative regulation of cell differentiation
negative regulation of cell activation
negative regulation of cellular component movement
positive regulation of locomotion
regulation of cell development
positive regulation of cellular component movement
regulation of cell motility
regulation of leukocyte activation
regulation of angiogenesis
regulation of chemotaxis
regulation of epithelial cell differentiation involved in kidney development
regulation of apoptotic process
negative regulation of programmed cell death
negative regulation of nervous system development
regulation of neurogenesis
negative regulation of cell development
positive regulation of cell motility
regulation of cell migration
regulation of apoptotic signaling pathway
regulation of nephron tubule epithelial cell differentiation
negative regulation of apoptotic process
regulation of epithelial cell apoptotic process
negative regulation of neurogenesis
regulation of neuron differentiation
positive regulation of cell migration
regulation of smooth muscle cell migration
negative regulation of apoptotic signaling pathway
regulation of extrinsic apoptotic signaling pathway
regulation of endothelial cell apoptotic process
negative regulation of neuron differentiation
P-value color scale
$> 10^{-3}$
10^{-3} to 10^{-5}
10^{-5} to 10^{-7}
10^{-7} to 10^{-9}
$< 10^{-9}$

Supplementary Figure S5 (on previous page). Gene Ontology enrichment analysis on differentially expressed genes between the Dex-treated MDA-MB-231 and MDA-MB-231-HM.LNm5 cell lines. RNA-Seq was performed on MDA-MB-231 and MDA-MB-231-HM.LNm5 cells which were serum-starved for 24 h then treated with dexamethasone (100nM) or vehicle control for 24 hours. Gene Ontology enrichment analysis was performed using GOrilla (http://cbl-gorilla.cs.technion.ac.il) on a hierarchical list of differentially expressed genes between the Dex-treated MDA-MB-231 and MDA-MB-231-HM.LNm5 cell lines. **A.** The entire Gene Ontology visualisation from GOrilla. **B.** A magnified section of the most highly enriched ontologies of differentially expressed genes.
